# NLRP3 Inflammasome and Gut Dysbiosis Linking Diabetes Mellitus and Inflammatory Bowel Disease

**DOI:** 10.26502/aimr.0178

**Published:** 2024-08-31

**Authors:** Ugljesa Malicevic, Vikrant Rai, Ranko Skrbic, Devendra K. Agrawal

**Affiliations:** 1 Department of Translational Research, Western University of Health Sciences, Pomona, California 91766, USA; 2 Centre for Biomedical Research, Faculty of Medicine, University of Banja Luka, Banja Luka, Republic of Srpska, Bosnia and Herzegovina; 3 Departments of Pathophysiology, Pharmacology, Toxicology and Clinical Pharmacology, Faculty of Medicine, University of Banja Luka, Banja Luka, Republic of Srpska, Bosnia and Herzegovina.

**Keywords:** Bile acids, Chronic inflammation, Crohn’s disease, Diabetes mellitus, Gut dysbiosis, Inflammatory bowel disease, NLRP3, Therapeutic targets, Toll-like Receptor, Ulcerative colitis

## Abstract

Diabetes mellitus and inflammatory bowel disease are chronic conditions with significant overlap in their pathophysiology, primarily driven by chronic inflammation. Both diseases are characterized by an aberrant immune response and disrupted homeostasis in various tissues. However, it remains unclear which disease develops first, and which one contributes to the other. Diabetes mellitus increases the risk of inflammatory bowel disease and inflammatory bowel disease may increase the risk of developing diabetes. This review focuses on comprehensively discussing the factors commonly contributing to the pathogenesis of diabetes mellitus and inflammatory bowel disease to draw a relationship between them and the possibility of targeting common factors to attenuate the incidence of one if the other is present. A key player in the intersection of diabetes mellitus and inflammatory bowel disease is the NLRP3 inflammasome, which regulates the production of pro-inflammatory cytokines leading to prolonged inflammation and tissue damage. Additionally, toll-like receptors via sensing microbial components contribute to diabetes mellitus and inflammatory bowel disease by initiating inflammatory responses. Gut dysbiosis, a common link in both diseases, further intensifies inflammation and metabolic dysfunction. Alterations in gut microbiota composition affect intestinal permeability and immune modulation, perpetuating a vicious cycle of inflammation and disease progression by changing protein expression. The overlap in the underlying inflammatory mechanisms has led to the potential of targeting mediators of chronic inflammation using anti-inflammatory drugs and biologics that benefit both conditions or attenuate the incidence of one in the presence of the other.

## Introduction

Diabetes mellitus (DM) is a chronic non-communicable metabolic disease typically marked by elevated blood glucose levels, commonly known as hyperglycemia [1]. The two main types of DM are Type 1 and Type 2. Diabetes mellitus type 1 (T1DM) is also called insulin-dependent diabetes mellitus (IDDM), while type 2 diabetes (T2DM) is known as non-insulin-dependent diabetes mellitus (NIDDM) [2]. Gestational diabetes (GDM) is a distinct type of diabetes that manifests itself for the first time during pregnancy. It is classified as a pre-type of T2DM and is characterized by a gradual decrease in β-cell function alongside an increase in insulin resistance (IR) [3]. Hyperglycemia may also be caused by medications and chemicals, disorders affecting the exocrine pancreas, monogenic diabetes syndromes, etc. [2]. The worldwide prevalence of DM among individuals aged 20–79 in 2021 was 10.5%, totaling 536.6 million people, and is forecasted to rise to 12.2% (783.2 million) by 2045 [4]. Diabetes rates were comparable between genders and peaked among those aged 75–79. According to their research, the prevalence of diabetes was higher in urban areas (12.1%) compared to rural regions (8.3%), and the trend was similar in high-income countries (11.1%) compared to low-income countries (5.5%). Middle-income countries are expected to witness the largest relative increase in diabetes prevalence (21.1%) between 2021 and 2045, surpassing high-income (12.2%) and low-income (11.9%) nations [4].

**Figure 1:**
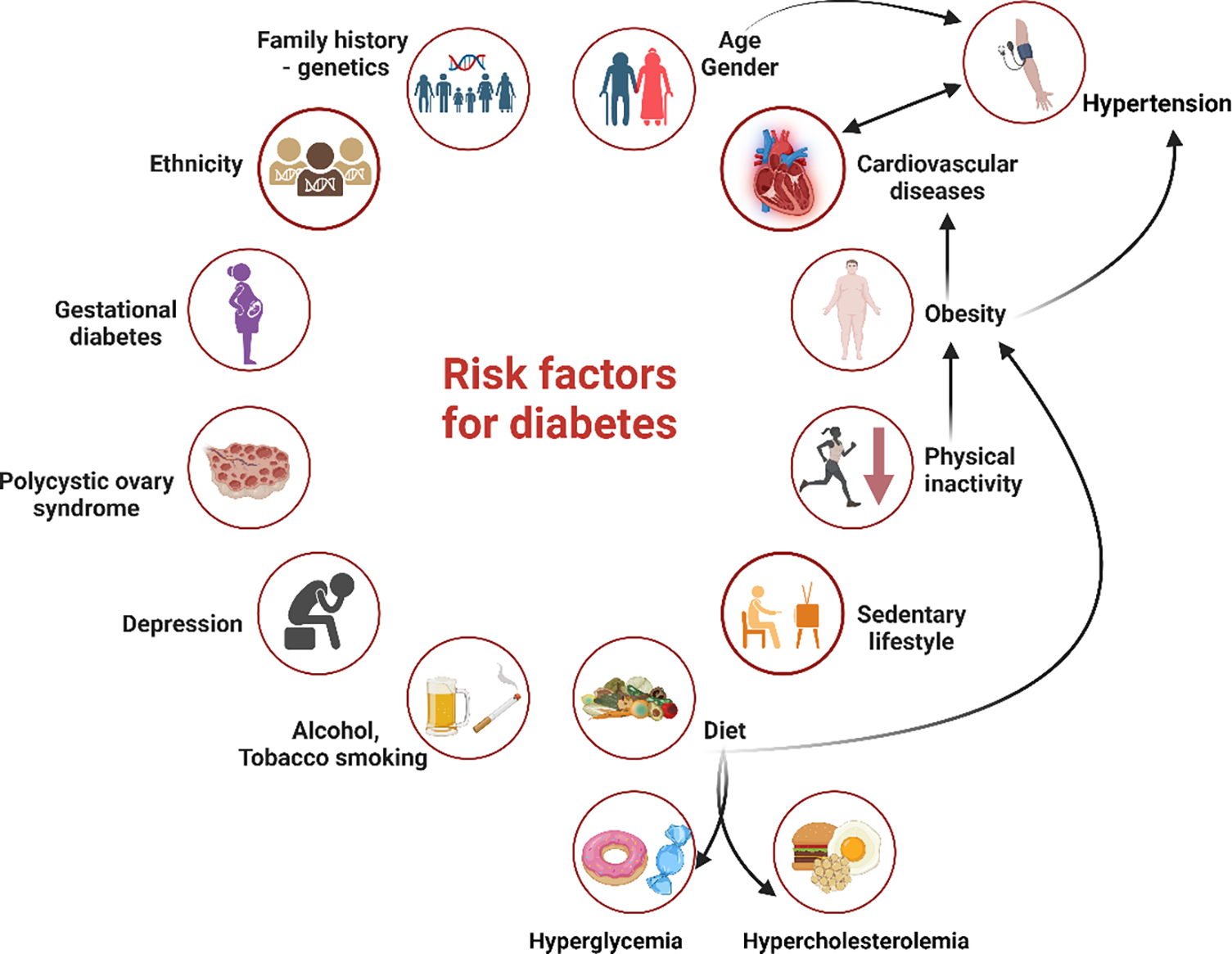
Risk factors for diabetes. T1DM and T2DM are the two primary forms of diabetes mellitus. The risk factors for T1DM include a family history, genetic predisposition, a specific ethnic background, and age. For T2DM, there are two categories of risk factors: modifiable and non-modifiable. Non-modifiable factors include family history/genetics, race or ethnic background, age, and gestational diabetes. Modifiable factors encompass dietary choices, alcohol and tobacco consumption, physical inactivity, obesity, cardiovascular diseases, polycystic ovary syndrome, depression, and others.

The associated healthcare costs of DM are significant, estimated at 966 billion USD in 2021, and projected to escalate to 1,054 billion USD by 2045 [5]. Given these immense human and financial burdens, as well as the great challenge of achieving effective post-diagnosis treatment, DM prevention has become imperative in modern medicine [4,5]. According to the World Health Organization, diabetes has shown a significant 70% rise since 2000, placing it among the top 10 causes of death. DM has experienced the most significant rise in male mortality among the top 10 causes, with an 80% increase since 2000 [6]. Reflecting on all this information, it is evident that we are confronting a global epidemic that presents one of the most important challenges of the 21st century since there are many risk factors for the initiation and progression of DM (Figure 1). Hyperglycemic states induce specific forms of DNA damage, peroxidation, and moderate stress in various tissues, particularly pancreatic islet β-cells, which are exposed to direct damage. Additionally, studies have shown that these very effects contribute to insulin resistance-mediated pathology [7,8]. Elevated blood glucose levels can also increase the production of free radicals. Exposure of pancreatic β-cells to oxidative stress can lead to a reduction in insulin gene expression. This is caused by inhibiting promoter activity and insulin mRNA expression [9]. Moreover, hyperglycemia is believed to induce oxidative stress, resulting in elevated levels of pro-inflammatory proteins and cytokines. Unmanaged diabetes can impact various organs and systems, resulting in a range of complications categorized as micro- and macrovascular [10].

The prevalent chronic complications that can significantly compromise health and even result in fatality include cardiovascular (hypertension, angina, chronic heart failure, myocardial infarction, and peripheral vascular disease), cerebrovascular (stroke and transient ischemic attack), ocular lesions (retinopathy, cataracts, and blindness), nephropathy (microalbuminuria, macroalbuminuria, renal hypofunction, and renal failure), diabetic foot problems, etc. [11]. However, it is crucial to recognize that diabetes generally affects all organ systems, including the gastrointestinal system. Epidemiological evidence indicates a link between metabolic illnesses such as DM and digestive disorders such as chronic constipation, which may be caused by delayed motility of the colon, enteropathic diarrhea, colorectal cancer, and inflammatory bowel disease (IBD) [12]. This article critically reviewed the correlation between two significant medical conditions, DM and IBD, and the underlying pathophysiology.

## Diabetes and Inflammation

Diabetes mellitus, type 1 and type 2, are both recognized as chronic diseases characterized by persistent inflammation. T2DM is the prevailing and most common type of diabetes, accounting for approximately 90–95% of all diagnosed cases. Insulin resistance is the most important factor in the pathophysiology of this type; however, a relative deficiency of insulin may have contributed to its development [2]. Nevertheless, it is important to note that not all individuals with insulin resistance will eventually develop T2DM. For instance, only approximately one-third of obese, insulin-resistant patients develop chronic hyperglycemia and T2DM [13].

The etiology of diabetes, its link to obesity, and the importance of adipose tissue are well-known today. Obesity is positively correlated with many illnesses, such as metabolic syndrome, diabetes, and dyslipidemia, characterized by chronic inflammation, which is a common and potentially underlying cause. These diseases are linked to elevated levels of inflammatory biomarkers, which may predict insulin resistance and T2DM [14]. They also trigger two major inflammatory pathways, known as stress-activated pathways, the Nuclear Factor kappa beta (NF-κB), mitogen-activated protein kinase (MAPK), and Jun N-terminal kinases (JNK), leading to the increased production of pro-inflammatory cytokines including IL-6, IL-1, and TNF-α from hypertrophic adipocytes [15,16,17], contributing to obesity-induced blocked insulin receptor activation, insulin resistance, and diabetes. Adipose tissue secretes TNF-α and a variety of bioactive substances known as adipokines, which are involved in inflammatory pathways. These substances include adiponectin, leptin, IL-1, IL-6, IL-10, angiotensinogen, chemokines, serum amyloid protein, and many more, which can contribute to the worsening of insulin resistance and T2DM [18]. When exposed to high concentrations of hyperglycemia and free fatty acids (FFA), pancreatic beta cells experience apoptosis; oxidative stress is the primary cause of this apoptotic response [19].

Glucose, as one of the primary cell stressors, can induce the generation of reactive oxygen species (ROS) because its elevated levels decrease the activity of antioxidants, therefore triggering and amplifying the production of inflammatory cytokines and chemokines [20]. Elevated levels of numerous acute-phase proteins, such as C-reactive protein and fibrinogen, as well as cytokines (IL-6, IL-1, and TNF-α) and chemokines, indicate T2DM [21]. Further, elevated levels of these cytokines secreted from adipocytes contribute to IBD [22], which in turn is associated with increased secretion of IL-6, IL-1, and TNF-α [23]. This suggests that obesity-induced inflammation may contribute to IBD and increased pro-inflammatory cytokines in IBD may also contribute to systemic inflammation and probably to insulin resistance; however, this relationship is not yet well understood.

## Inflammatory Bowel Disease

**Figure 2:**
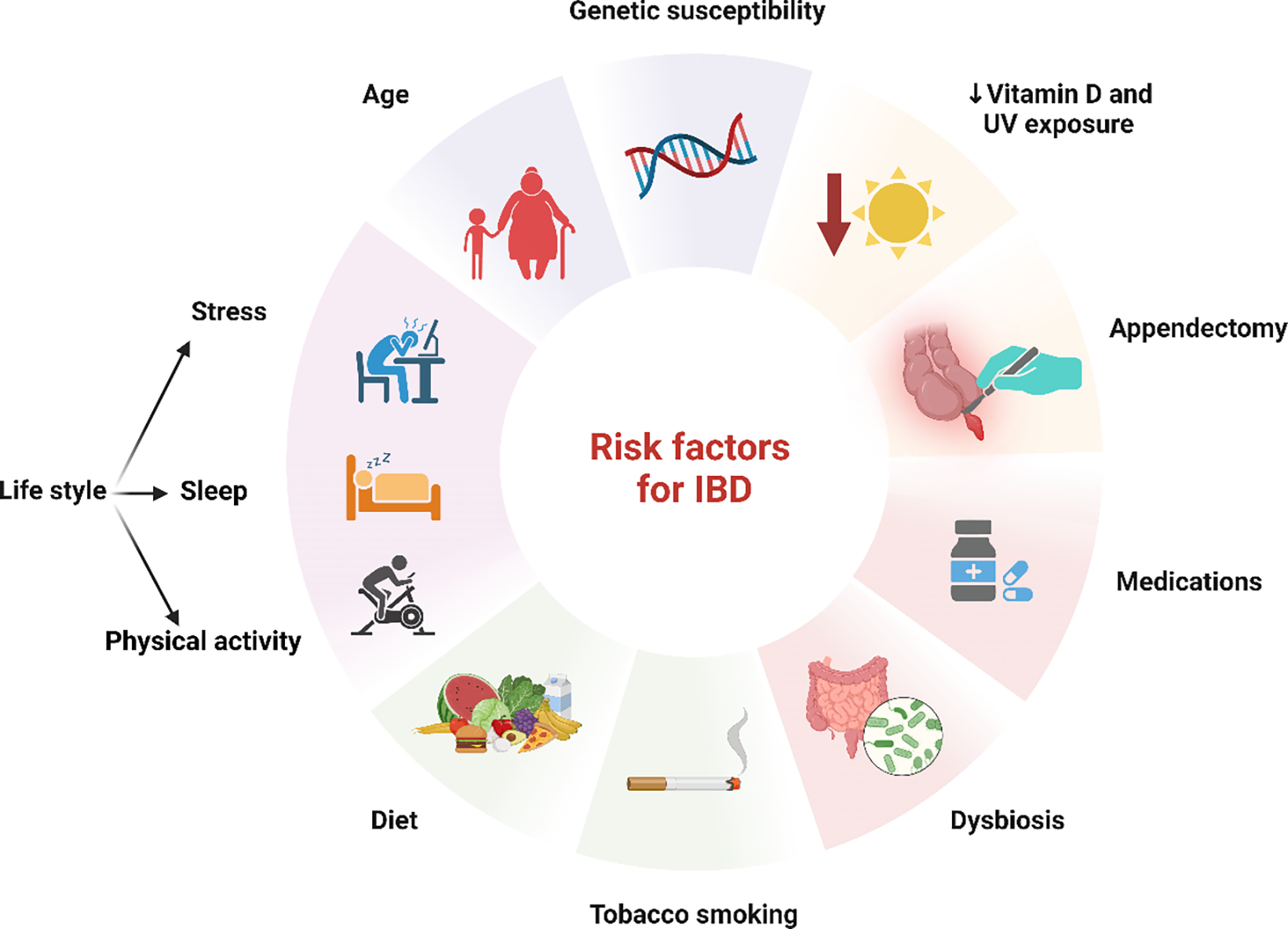
Risk factors for Inflammatory Bowel Disease. Risk factors for IBD include genetic predisposition, age, and lifestyle factors such as chronic stress, lack of sleep, and physical inactivity. Unhealthy habits like a high-fat, low-fiber diet, and smoking significantly elevate risk, particularly for Crohn’s disease. Other important contributors to IBD are dysbiosis, vitamin D deficiency, appendectomy, and the use of various medications. These factors, combined with genetics, drive the onset and progression of IBD.

Inflammatory bowel disease (IBD) encompasses a variety of conditions marked by persistent inflammation within the gastrointestinal (GI) tract, remarkably including Crohn’s disease (CD) and ulcerative colitis (UC) [24]. Although CD and UC both exhibit chronic inflammation, the precise pathogenic connection between these disorders remains unclear. While many factors are known to play a role, researchers believe that an imbalance in the homeostasis of the luminal mucosa in genetically predisposed individuals could be a potential primary trigger [25,26]. Moreover, it is considered that IBD exhibits close associations with smoking, dietary factors, nonsteroidal anti-inflammatory drugs (NSAIDs), and vaccination [27,26]. Family history and age are also some of the most important risk factors for the occurrence of this disease (Figure 2).

The prevailing trend in IBD development indicates that most individuals are diagnosed before reaching 30 years of age [28]. However, it is important to note that some people may not manifest signs of the disease until their 50s or 60s and are diagnosed in their 60s [29]. Patients with IBD may exhibit a range of symptoms, which can vary between UC and CD. However, their typical signs include ongoing abdominal discomfort, persistent diarrhea (with or without bleeding), fatigue, and unintentional weight loss [30]. Aside from the expected effects on the intestines, this disease also has a wide range of extraintestinal symptoms, such as anemia, myocarditis, pulmonary disorders, hepatobiliary complications, bone demineralization, and effects on the skin, eyes, and endocrine systems. These symptoms make the disease’s diagnosis much more difficult. Delayed extra-intestinal manifestations (EIM) may still occur later in the disease course, impacting life and healthcare resources [31]. However, the chronological multinational study findings showed that, while the average age of the first presentation of IBD has not changed during the last 2 decades, the rate of extraintestinal involvement has decreased during the last 5 years [32]. Many recent studies have focused on elucidating the correlation between DM and IBD [33,34,35]. This has not always been the case historically, some researchers have suggested that these two diseases do not significantly influence each other’s occurrence [36,37]. However, the question arises whether IBD is a risk factor for the development of T2DM or vice versa? Villumsen et al. reported in their research that IBD represents a high risk for T2DM incidence. On the other hand, Abrahami et al. discovered that using dipeptidyl peptidase-4 inhibitors (DPP4i), a type of oral diabetic medication therapy in T2DM, can cause IBD, but another study presented contradictory findings, showing that DPP4i use in T2DM therapy could reduce the risk of IBD [38,39,40].

Overall, the findings suggest that T2DM and IBD share common risk factors such as genetic predisposition, gut bacteria, and lifestyle, providing a closer link between these two clinical entities [33]. Therefore, understanding the possible cause-and-effect link between IBD and DM could help us understand different biological processes and come up with better and more effective preventive strategies [41].

## Link between Diabetes Mellitus and Inflammatory Bowel Disease

There may be a common mechanism, including hereditary factors and chronic inflammation, that underlies the relationship between DM and IBD. It is thought that there exist 10 genetic loci that have been linked to both IBD and T1DM. Genetic variations partially influence the susceptibility to both IBD and T1DM at certain sites, including PTPN2, ORMDL3, IL2/IL21, IL2RA, IL10, IL18RAP, BACH2, TYK2, IL27, and PTPN22. T2DM is associated with five common genetic locations: HNF4A, CAPN10, CDKAL1, THADA, and GCKR [42,43,44]. Tang et al. performed a comprehensive bidirectional two-sample Mendelian randomization (MR) analysis in individuals of European descent to investigate the association between IBD and T2DM. Nevertheless, no strong evidence indicates a link between an individual’s genetically determined IBD and T2DM [41]. Xu et al. conducted a similar study that showed a correlation between T2DM and a decreased risk of UC. However, no substantial causal connections were discovered between T2DM and CD, UC and T2DM, or CD and T2DM [45]. Applying the same approach to examine the cause-and-effect relationships between IBD and other risk variables, Saedh et al. could not find any correlation to previous studies. Although they observed a possible link between T2DM risk and CD, this correlation was no longer significant when alcohol use was considered during the study [46]. Nevertheless, Chen et al. discovered a decreased probability of developing UC in their MR study. They also discovered a significant correlation between a lower incidence of Crohn’s disease and genetically determined fasting glucose levels [47].

Indeed, DM and IBD are complex conditions that arise from a variety of factors. Both are associated with chronic inflammation and an imbalance of microorganisms in the gastrointestinal tract. As a result, numerous pro-inflammatory compounds and associated molecules are expressed more frequently, either locally or systemically. Some authors suggest that imbalance might be the molecular bridge connecting different inflammatory pathways, including TGFβ, NF-κB, TNF-α, and reactive oxygen species (ROS) [48]. TGF-β plays a crucial role in the malfunction of β cells in the pancreas, which leads to DM development. Glucotoxicity activates the TGF signaling pathway, leading to dysfunction and the subsequent production of reactive oxygen species (ROS) in the pancreatic islets. The insufficient presence of anti-oxidative enzymes in β cells leads to the accumulation of ROS, which can cause oxidative stress, a known trigger of β cell apoptosis [48]. Moreover, it is thought that the oxidative stress brought on by hyperglycemia raises the number of cytokines like IL-1β, TNFα, IL-6, and IL-10 that promote inflammation [49,14]. TGF-β receptors are found in many types of cells, including immune cells and epithelial cells. They play different roles in keeping the immune system in balance in the intestines [50]. In several animal models, mice that did not have certain TGF-β signaling pathways turned on in their T-cells developed severe systemic autoimmunity and spontaneous severe colitis very quickly. Systemic autoimmunity was marked by notable lymphocyte infiltration and activated T-cells across multiple organs [51].

As previously noted, interleukin 6 (IL-6) is considered one of the pivotal cytokines in the pathogenesis of DM. According to Rehman et al., IL-6 is a pro-inflammatory cytokine that influences cellular processes such as differentiation, migration, proliferation, and cell death, in addition to inducing inflammation. These effects are linked to insulin resistance and the pathophysiology of T2DM [52]. Furthermore, it regulates the T cell activation and differentiation process, inhibiting the proliferation of regulatory T cells and regulating migration, proliferation, and cell death. In tandem with TGF-β, it promotes the development of naive T cells into Th17 cells, a subpopulation of T helper cells that generates the inflammatory cytokine IL-17 [52,53]. The Th17 cell subset is thought to have an important effect on the development of IBD and can initiate a carcinogenic process if inflammation is not controlled appropriately [54]. IL-17-secreting Th17 cells have been identified as playing a role in the development of T1DM via increased inflammation. They inhibit the actions of Treg cells, which support immunological tolerance, attract and activate neutrophils and macrophages, and boost the production of pro-inflammatory cytokines and chemokines. This suggests that IL-17 mainly enhances inflammatory reactions, which leads to the autoimmune death of β-cells that produce insulin in the pancreas [55]. Furthermore, IL-17 contributes to T2DM progression by activating the NF-κB pathway, which increases the production of genes that produce pro-inflammatory cytokines. These cytokines have been identified as disrupting the insulin signaling process, resulting in insulin resistance and the onset of T2DM [56,55]. IL-17 induces the expression of chemokines such as IL-6, whose role we have explained, and TNF-α [53]. TNF-α is a crucial pro-inflammatory agent that greatly contributes to the onset of insulin resistance and the development of T2DM. The process occurs by inducing mild inflammation in specific tissues by activating different transcriptional-mediated molecular pathways, particularly IKKβ, JNK, and NF-κB [57]. Individuals with IBD exhibit increased levels of TNF-α in the mucosa, highlighting its crucial involvement in developing the disease.

TNF-α also initiates other signaling pathways, such as NF-kB and the Mitogen-Activated Protein Kinases (MAPK) pathway, which increase the activity of activator protein 1 (AP-1) and Jun N-terminal kinase (JNK) [58]. AP-1 plays a critical role in the pathogenesis of IBD by inducing inflammation and has been considered a therapeutic target. Activation of AP-1 triggers adaptive changes in injured cells, including the production of pro-inflammatory mediator genes and apoptosis, specifically targeting genes like IL-1b and TNF-α [59]. TNF-α, on the other hand, can activate AP-1, leading to the production of tissue remodeling proteases like collagenase and pro-inflammatory cell adhesion molecules like E-selectin [60]. Both soluble (sTNF-α) and membrane-bound (mTNF-α) and other pro-inflammatory cytokines like interleukin (IL)-1β, IL-6, and IL-18 are produced by different stromal and immune cells in the inflamed mucosa. Interestingly, non-immune cells also create pro-inflammatory cytokines. For example, stromal fibroblasts produce TNF-α and IL-6, whereas intestinal epithelial cells produce IL-18 and other members of the IL-1 cytokine family. TNF-α has numerous pro-inflammatory effects via its receptors, tumor necrosis factor receptor 1 (TNFR1) and tumor necrosis factor receptor 2 (TNFR2). These effects include activating macrophages and effector T cells, causing Paneth cell death, boosting angiogenesis, and encouraging the synthesis of matrix metalloproteases [61]. The sTNF-α binds specifically to the TNFR1 receptor, starting a series of signals that cause cell division, apoptosis, and the release of cytokines [62]. On the contrary, mTNF can attach to both TNFR1 and TNFR2. It was observed that TNFR2 expression is more restricted than TNFR1 expression. According to Yang et al. (2018), this means that the sTNF-α-mediated signaling pathway through TNFR1 mainly causes inflammation, while mTNF-α binding to TNFR2 mainly starts tissue regeneration and immune system changes [63]. Both DM and IBD patients have high expression levels of these receptors, which contributes to the development and course of these two illnesses. TNF-α has a significant role in the development of diabetes, particularly T2DM. Increased TNF-α impairs insulin signaling by serine phosphorylation, which causes IR in adipocytes and peripheral tissues and eventually results in T2DM [57].

The gut, particularly intestinal epithelial cells (IEC), is important in maintaining immunological and metabolic homeostasis [64]. Most gut epithelial cells are secretory goblet and Paneth cells, which produce antimicrobial peptides and absorb nutrients. Furthermore, there is a distinct and specialized category of cells called enteroendocrine cells, which release over 20 distinct peptide hormones, including glucagon-like peptides 1 and 2 (GLP-1, GLP-2), glucose-dependent insulinotropic peptide (GIP), etc. [65]. Intestinal endocrine L cells, primarily found in the ileum and large intestine (colon), release GLP-1, which can also regulate the proliferation, programmed cell death, and differentiation of β cells, thereby improving their function. Consequently, a decrease in GLP-1 production can lead to insulin resistance and contribute to the development of T2DM [66]. In conditions like IBD, various cytokines cause persistent intestine inflammation. This results in increased permeability of the intestinal barrier and an imbalance in the gut flora, with macrophages secreting a range of cytokines, such as TNF-α and the IL-1 family of cytokines (IL-1α, IL-1β, IL-18, IL-33, IL-36, and IL-37), IL-6, IL-12, IL-23, etc. [67]. L cells express TNFR1, and long-term exposure to TNF-α has been shown to hinder the production of GLP-1, which leads to IR and T2DM [68].

## NLRP3 Inflammasome

The cytoplasm of active immune cells, such as lymphocytes, monocytes, macrophages, and dendritic cells, contains large intracellular multiprotein complexes known as inflammasomes. They are thought to play a crucial role in the development of many inflammatory disorders in humans, including diabetes, obesity, heart diseases, cancers, and autoimmune diseases like IBD, rheumatoid arthritis, multiple sclerosis, etc. [69]. The NOD-like receptor family, pyrin domain-containing (NLRP), are immune system receptors that contain several subtypes, with NLRP3 being the most extensively researched and one of the most important inflammasomes [70]. Upon activation of the NLRP3, it triggers the process of pro-caspase-1 self-cleavage and engagement, leading to the generation of the pro-inflammatory cytokines, interleukin 1β (IL-1β) and interleukin 18 (IL-18) [71]. NLRP3, pro-IL-1β, and pro-IL-18 are all triggered by recognizing Pathogen-Associated Molecular Patterns (PAMPs), molecules released by damaged cells called the Damage-Associated Molecular Patterns (DAMPs), pattern recognition receptors like toll-like receptors (TLRs), or cytokines like TNF-α [72]. Elevated glucose levels have long been known to activate the NLRP3 inflammasome. TLRs are believed to play a key role in T1DM pathogenesis. Their activation triggers the NLRP3 to produce IL1β, suggesting that this cytokine mediates the effects of TLRs on T1DM [73]. On the other hand, the role of NLRP3 in the development of IR and T2DM is much clearer. When islet cells are exposed to chronic hyperglycemia, they will trigger the activation of NLRP3 and the release of IL-1β and IL-18 [74]. The activation of NLRP3 and the subsequent generation of IL-1β were initially seen in islet-infiltrating macrophages and pancreatic β-cells. It was shown that IL-1β enhances glucose absorption into macrophages; insulin amplifies the pro-inflammatory effects by controlling the insulin receptor, glucose metabolism, and ROS generation, causing oxidative as well as ER stress [75]. IL-18, also referred to as an interferon-gamma-inducing factor, stimulates the production of TNF-α, which in turn promotes the generation and release of IL-6 and CRP, contributing to inflammation [76].

Obesity stimulates the production of NLRP3 in diabetic patients, and there is a strong correlation between obesity and increased NLRP3 expression in adipose tissue and IR. Numerous immune cells, particularly pro-inflammatory macrophages, increase the production of cytokines including TNF-α, IL-1β, and IL-6, which in turn affects the homeostasis of adipose tissue. NLRP3 activation appears to significantly regulate adipocyte differentiation, leading to increased IR in adipocytes [77,78]. In maintaining gut homeostasis, NLRP3 plays an important role in host defense by regulating the integrity of intestinal epithelial cells and the immune system’s response to the gut microbiota [79]. Alterations in the gut microbiota contribute to IBD. NLRP3 activation alters the composition of the gut microbiota, while short-chain fatty acids (SCFAs) and other gut bacteria byproducts help G protein-coupled receptors (GPCRs) cause NLRP3 activation [80]. Consequently, its activation leads to the overproduction of IL-1β, leading to immune responses in the mucosa, T cell proliferation, and neutrophil recruitment via IL-1β and IL1R complexes [81]. This activates the NF-κB and MAPK pathways, resulting in increased production of pro-inflammatory cytokines such as IL-6, IL-8, and TNF-α [82]. Increased secretion of these cytokines mediated persistent inflammation, a risk factor and underlying etiology for IBD. When it comes to IL-18, some studies showed that caspase-1 is required for epithelial IL-18 production but not NLRP3. On the other hand, NLRP3 is essential to caspase-1 activation and may also contribute to intestinal IL-18 synthesis [83]. Increased IL-18 production may increase chronic inflammation and tumor growth by activating IFN-γ and promoting the Th1 response. IL-18 triggers IFN-γ release by Th1 and NK cells, impacting intestinal development and repair after epithelial injury. It remains a mystery whether greater IL-18 levels in lamina propria mononuclear cells and intestinal epithelial cells in IBD patients may trigger or worsen IBD pathogenesis [84].

## Toll-Like Receptors in Diabetes Mellitus and Inflammatory Bowel Disease

**Figure 3:**
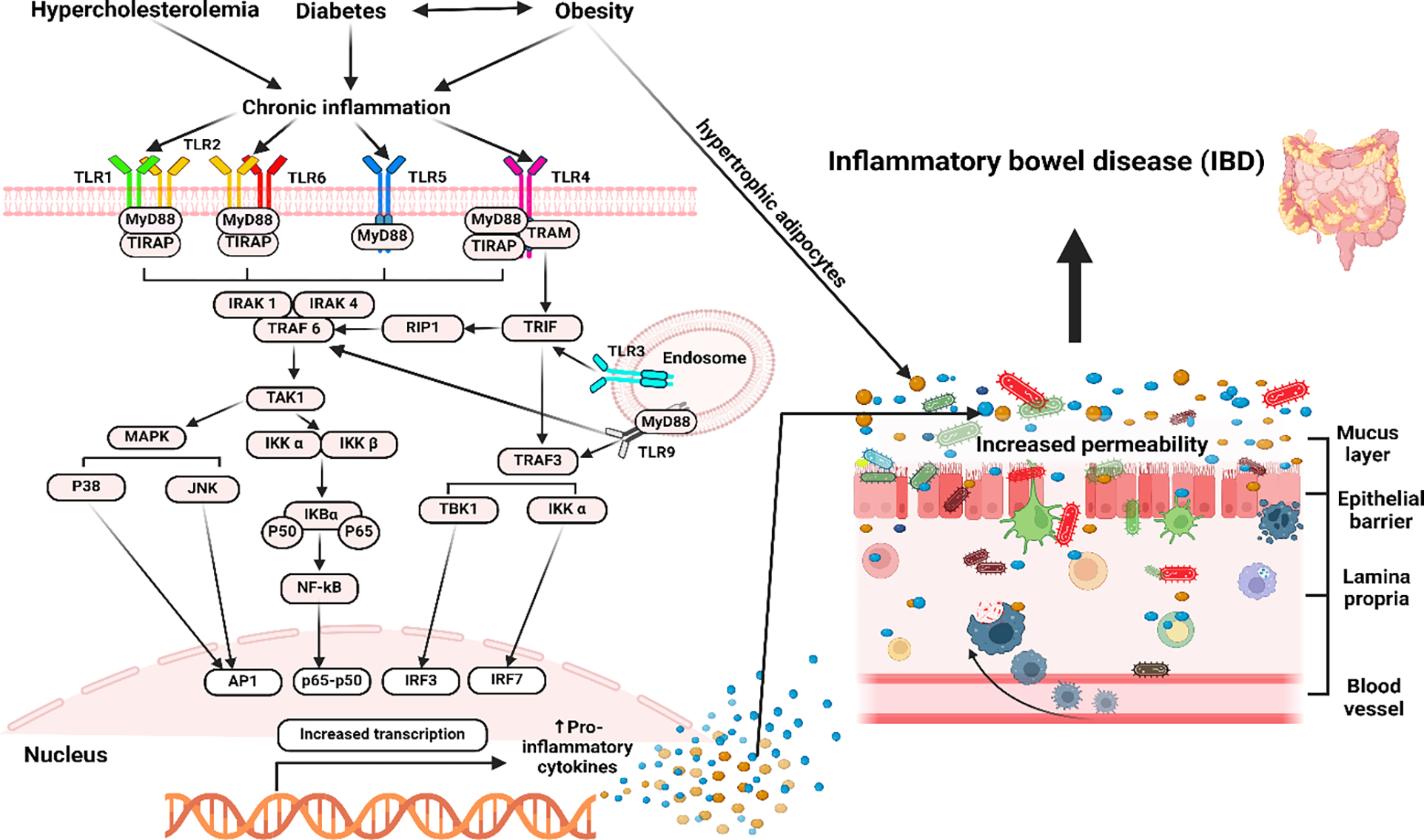
The illustration above highlights the role of TLRs and the process of their activation in DM and its subsequent correlation with IBD. Diabetes, especially T2DM, and obesity are characterized by persistent low-grade inflammation, which plays a crucial role in the etiology and development of these conditions. Hyperglycemia, high FFA levels, oxidative stress, and other conditions trigger mostly TLR2 and TLR4 activation, which in turn promotes the creation of several transcriptional factors that lead to the generation of pro-inflammatory cytokines and inflammation. Inflammation and the subsequent production of cytokines in the gut result in dysbiosis, which leads to increased permeability of the intestines, sometimes referred to as “leaky gut” by impairing epithelial tight junctions and facilitating the translocation of microbial products like lipopolysaccharide (LPS) into the bloodstream, which in turn causes systemic inflammation. Increased concentrations of circulating LPS are linked to the development of insulin resistance. Dysbiosis can initiate abnormal immunological reactions, resulting in an excessive release of pro-inflammatory cytokines that can contribute to the development of IBD; therefore, implying a connection between diabetes, obesity, and IBD.

Toll-like receptors (TLRs), a family of pattern recognition receptors (PRRs), are a crucial group of receptors that provide the initial defense mechanism against a wide range of pathogens [85]. Mammals contain a total of 13 TLRs (TLR1–13), with 10 of these found in humans (TLR1–10) [86]. A certain subset of TLRs is present in every immune system cell, and they have unique roles in modulating immune responses and identifying exogenous PAMP and endogenous DAMP ligands. The interaction between the ligand and TLR/TLRs leads to its activation, resulting in the transmission of signals into the cell via molecules such as myeloid differentiation primary response 88 (MyD88), TRIF-related adapter molecule (TRAM), TIR-domain-containing adapter-inducing interferon-β (TRIF), etc. [87]. The activation of specific intracellular downstream signaling cascades by ligand binding to TLRs results in the engagement of at least two different pathways (Figure 3). All TLRs, except TLR3, use the MyD88-dependent pathway, while TLR-3 and TLR-4 use the TRIF-dependent system. MyD88 primarily triggers the activation of NFκB family members and MAPK, generating pro-inflammatory cytokines. On the other hand, TRIF mostly activates IRF family members and tends to promote an anti-viral response by inducing interferon [88]. Activation of TLR-mediated downstream signaling results in increased secretion of pro-inflammatory cytokines, which play a critical role in the pathogenesis of both DM and IBD [14,89]. Further, TLR-2, TLR-4, TLR-5, TLR-9, and others play a critical role in the pathogenesis of IBD [90,91], and TLR-2, TLR-1, TLR-4, TLR-6, and TLR-7 contribute to diabetes [92,93]. This suggests that TLRs are a common contributing factor to DM and IBD by inducing chronic inflammation (Figure 3).

## Dysbiosis in Diabetes Mellitus and Inflammatory Bowel Disease

In healthy individuals, the gut microbiota plays a major role in many physiological metabolic processes, including nutrition, immunity, metabolism, and pathogen defense [94]. Nevertheless, recent data suggests that alterations in its composition, referred to as dysbiosis, may play a role in the onset of metabolic disorders such as DM, cardiovascular diseases, IBD, and colorectal cancer [95]. Dysbiosis-mediated development of diabetes is caused by increased intestinal permeability, leading to dyslipidemia and persistent inflammation in the body [96]. Due to the impaired function of the gut barrier, gut bacteria can have close contact with the gut epithelium, promoting immune cell infiltration, pro-inflammatory cytokine expression, and oxidative stress, releasing lipopolysaccharide (LPS) [96]. The gut microbiota of diabetic patients includes higher concentrations of pathogenic bacteria, including Prevotella copri and Bacteroides vulgatus, as well as different Enterobacteriaceae, Clostridiales, Escherichia coli, and Lactobacilli [95]. Bacteroides play a primary role in the production of LPS in the human gut microbiome [97]. Consequently, LPS produces pro-inflammatory cytokines such as TNF-α, IL-1, IL-6, and iNOS, which can lead to subclinical pro-inflammatory conditions and the development of IR and T2DM [95]. Upon initiating the inflammatory cascade, activating serine kinases, such as JNK and IKK, can lead to serine phosphorylation of the insulin receptor substrate (IRS), leading to insulin signaling inhibition and cellular insulin resistance. Further, increased blood sugar levels can also damage the tight and adherent junctions in the intestinal wall of streptozotocin-induced diabetic mice model [98]. This suggests that elevated blood sugar levels may enhance intestinal permeability, facilitating the passage of bacteria and chemicals that promote inflammation and disrupt glucose metabolism [99].

Increased intestinal permeability, decreased intestinal barrier function, and inflammation underlie IBD [100]. This suggests that impaired intestinal permeability and chronic inflammation in DM may contribute to IBD because IBD patients also showed a decrease in the level of bacteria with anti-inflammatory properties and an increase in bacteria with inflammatory properties compared to healthy individuals. A recent study examined the gut microbial composition at various taxonomic levels in healthy control individuals with active CD, UC, and ischemic colitis (IC). The study found a significant rise in the quantity of Latilactobacillus sakei and Enterococcus faecium in patients with CD, Ligilactobacillus ruminis and Enterococcus faecium in patients with UC and Enterococcus faecium, Escherichia coli, and Enterococcus faecalis in patients with IC that could serve as biomarkers for CD, UC, and IC, respectively [101]. The presence of most of them in dysbiosis associated with diabetes as well as dysregulation of tight proteins increasing intestinal permeability and resulting in impairment of the intestinal mechanical barriers suggest their critical role in the development of IBD [102]. Furthermore, growing evidence shows that intestinal epithelial cells that have undergone glycosylation exhibit more shortened O-glycans and different terminal glycan structures. Changes in glycan composition can damage the immune system and mucosal layer, ultimately leading to this disease [103]. The process of gut bacterial fermentation of dietary fiber abundantly generates SCFAs, which appear to play a crucial role in maintaining good health [104]. SCFAs manage the immune response in the mucosal lining by stimulating B cell growth and regulatory T cell (Treg) formation and proliferation. Additionally, SCFAs may trigger the release of inflammatory cytokines [105]. The marked decrease in their levels is one possible contributing factor to intestinal and immunological homeostasis disruption in IBD patients [106].

Dysbiosis and increased immune cell infiltration in IBD individuals may lead to a decrease in SCFAs, which in turn reduces the levels and functionality of Treg and increases the activity of neutrophils, macrophages, and dendritic cells [105,106]. Further, decreased SCFAs levels in the gut are associated with obesity and T2DM [107], and thus a decreased level of SCFAs in the gut in diabetic patients may contribute to IBD. The gut microbiota produces hydrogen sulfide (H2S), which is considered a cytoprotective metabolite at concentrations below 1 mM [108]. It triggers certain cellular anti-inflammatory responses, such as preventing caspase activation and apoptotic cell death, inhibiting leukocyte adhesion to the vascular endothelium, thereby reducing the infiltration of neutrophils and lymphocytes, inducing the expression of cyclooxygenase-2 (COX-2), and promoting neutrophil apoptosis [109,110]. The increased production of H2S by the gut flora provides a stronger explanation for the pathophysiology of IBD, either by an increased presence of H2S-producing bacteria or a malfunctioning H2S-detoxification system. When H2S levels are too high, it can change genes that control DNA repair, the cell cycle, and inflammatory responses. This makes it a genotoxic and cytotoxic agent for colonocytes [111]. H2S primarily causes cytotoxicity by inhibiting cytochrome C oxidase activity, an essential enzyme for the last stage of mitochondrial respiration. Therefore, inhibiting the oxidation of essential metabolites, including n-butyrate, L-glutamine, and acetate, lowers the cell’s bioenergetic efficiency. Further, an association of increased H2S with the development of T2DM due to the inhibition of insulin production via KATP channel activation results in increased circulating glucose, leading to progressive β-cell toxicity [112]. This suggests that H2S is a common etiological factor for DM and IBD.

## The role of Bile Acids in Diabetes Mellitus and Inflammatory Bowel Disease pathogenesis

Bile acids (BAs) are essential for several important physiological functions, such as digestion, glucose-lipid metabolism, and signaling pathways. Their role in developing DM and IBD has gained increasing attention, particularly due to their involvement in the gut-liver axis, metabolism, and inflammation [113]. A crucial factor in the development of both DM and IBD is gut dysbiosis [114,115], which significantly impacts BA metabolism. Since the key function of the gut microbiota is the transformation of primary into secondary BAs, any disruption of microbial balance leads to changes in the composition and activity of BA-metabolizing bacteria.

These acids are thought to have a diverse role in glucose metabolism and intestinal inflammation by engaging various important receptors, such as the farnesoid X receptor (FXR) and the Takeda G-protein-coupled receptor-5 (TGR5) [116]. FXR is primarily expressed in the liver, intestine, kidney, adipose tissue, and at lower levels in pancreatic β-cells. With a high binding affinity, chenodeoxycholic acid (CDCA) is the most effective BAs activator of FXR compared to deoxycholic acid (DCA), lithocholic acid (LCA), and cholic acid (CA) [117]. The variation in binding affinities among BAs affects their ability to modulate FXR-mediated signaling pathways, thereby influencing physiological processes such as BAs homeostasis and glucose and lipid metabolism. Upon binding BAs to FXR, activation of FXR in the liver results in increased glycogen synthesis and the downregulation of critical enzymes involved in gluconeogenesis [118]. This process reduces hepatic glucose production, which in turn lowers blood glucose levels. Although FXR expression in pancreatic β-cells is low, it can still significantly modulate insulin secretion. Activation of FXR in these cells influences insulin release by enhancing insulin secretion in response to glucose, thereby contributing to the regulation of blood glucose levels [119]. In the intestines, FXR is essential for maintaining intestinal barrier function by modulating the expression of tight junction proteins and enhancing mucosal defense, which is crucial for preventing inflammation and preserving the barrier’s integrity [120]. It was reported that patients with DM can exhibit increased levels of both primary and secondary BAs in plasma, consequently affecting FXR activation [121]. Increased BAs levels can lead to altered FXR signaling, impacting glucose homeostasis and insulin sensitivity, and contributing to the progression of this metabolic disease [122,123]. In IBD, although there is an increase in primary BAs, including CDCA, intestinal FXR activity was found notably reduced [124]. This indicates that despite elevated levels of these BAs, the regulatory function of FXR, which is crucial for maintaining BA homeostasis, modulating inflammation, and integrity of the intestinal barrier, is impaired in IBD patients [125]. Another critical BA receptor, TGR5, is expressed in various tissues, including the liver, intestines, adipose tissue, skeletal muscle, brain, and pancreas. TGR5 is broadly activated by various BAs, with LCA being the most potent activator. Activation of TGR5 stimulates the release of GLP-1 from enteroendocrine L-cells, subsequently promoting insulin secretion from pancreatic β-cells, improving insulin sensitivity, and helping blood glucose regulation [126]. In DM, dysregulation of secondary BAs can compromise these beneficial effects, potentially exacerbating this condition’s metabolic and inflammatory aspects. In the context of IBD, the deficiency of secondary BAs in the colon can significantly impair gastrointestinal motility through TGR5 signaling [127]. Additionally, impaired TGR5 activation in the intestinal mucosa has been demonstrated to exert pro-inflammatory effects by influencing macrophage function. Specifically, its impaired and reduced activation in macrophages results in the promotion of NF-κB signaling, consequently leading to increased production of pro-inflammatory cytokines [128]. Since BAs are critical regulators of glucose metabolism and inflammation primarily through their interaction with these two key receptors, any imbalance or disruption in their composition can impair these regulatory mechanisms and contribute to the development of DM and IBD.

Considering the function of BAs and their receptors, targeting them holds significant promise for improving DM and IBD. By enhancing receptor activation through pharmacological agents or modifying BA profiles, it is possible to reduce intestinal inflammation and improve insulin sensitivity, glucose regulation, and overall metabolic health. Although CDCA is a potent FXR activator, its therapeutic use is limited due to its hepatotoxicity and elevated transaminase levels [129]. In contrast, ursodeoxycholic acid (UDCA), a hydrophilic BA, is associated with lower liver toxicity and has shown positive effects in recent clinical studies. Despite its minimal FXR activation, Lakic et al. demonstrated the beneficial effects of UDCA treatment in patients with T2DM. It has been found to improve liver function, diastolic blood pressure, and oxidative stress markers. These effects suggest that UDCA could play a role in attenuating the progression and complications of diabetes, offering potential therapeutic benefits [130]. Wang et al. have reported that UDCA combined with mesalazine had positive effects in treating patients with UC. Regulation of the IL-23/IL-17 axis may be one of the mechanisms of these effects, along with UDCA’s positive role in the balance of intestinal microflora in patients with UC [131]. In the rat model, Milivojac et al. found that pretreatment with UDCA significantly attenuated both systemic and hepatic inflammation induced by LPS, demonstrating its anti-inflammatory properties. This intervention resulted in noticeable improvements in oxidative stress, reductions in serum levels of pro-inflammatory cytokines, and decreased expression of NF-κB in hepatocytes. Additionally, it resulted in favorable alterations in serum biochemical markers, including creatine kinase, lactate dehydrogenase, and high-sensitivity troponin I [132]. Targeting TGR5 can improve glucose metabolism by enhancing GLP-1 secretion and increasing insulin sensitivity [133]. Additionally, it can reduce inflammation by decreasing NF-κB activation and lowering the production of pro-inflammatory cytokines, contributing to TGR5’s role in managing DM and IBD [134]. Overall, while targeting BAs and their receptors holds promise for improving the management of both DM and IBD, it presents challenges including variability in patient responses, potential side effects, and an incomplete understanding of underlying mechanisms. These challenges underscore the importance of additional research to thoroughly understand the benefits and risks and to develop more effective therapeutic strategies.

## Therapy

**Figure 4:**
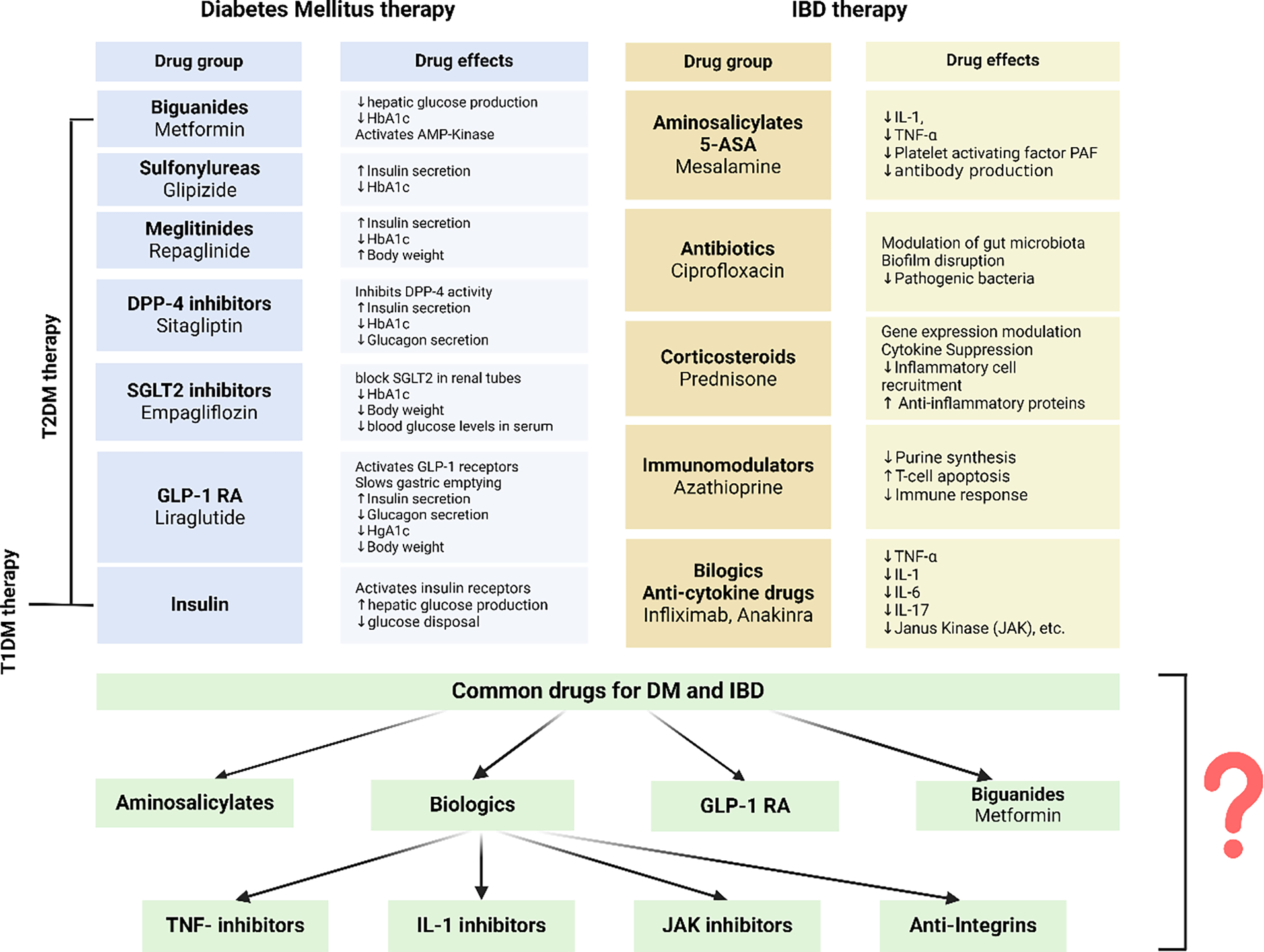
Groups of medicines used in the treatment of diabetes mellitus and inflammatory bowel disease, as well as groups of medicines that could potentially be used to treat individuals with both diabetes mellitus and inflammatory bowel disease. These drugs are shown with question mark (?).

Diabetes mellitus and inflammatory bowel disease share some similar underlying pathogenesis, with inflammation being the most significant. This implies that there could be a shared therapeutic approach for DM and IBD, however, this arena is not well understood or discussed. We have discussed the common therapeutics for DM and IBD in this section, as well as the probability of drugs that can be effectively used in both conditions (Figure 4).

In addition to the conventional and well-established therapy for these two diseases, it is noteworthy that both conditions employ certain medications that significantly enhance the clinical presentation. Aminosalicylate medications, as a type of disease-modifying antirheumatic drug (DMARD), are the cornerstone of treatment for active mild IBD, as they are often helpful in achieving remission in moderate versions of both disorders, CD and UC, especially in UC [135]. Further, high salicylate dosages can improve lipidemia, inflammatory-associated markers, and glucose metabolism in T2DM patients [136]. Goldfine et al. conducted a trial to assess the impact of salicylate versus placebo as an additional treatment for individuals with inadequately managed, established T2DM, specifically focusing on its effects on blood sugar levels. Throughout the research, salicylate effectively decreased both HbA1c and fasting blood glucose levels. Their study also confirmed lower levels of circulating white blood cells, neutrophils, and lymphocytes, showing that salicylate can help reduce inflammation [137]. Since cytokines are the main drivers of inflammation, focusing on them presents a promising approach to addressing different inflammatory diseases. Therefore, the use of biologics and immunomodulatory therapy can help reduce inflammation, but it is still unclear whether this form of therapy can improve the clinical outcome in individuals with both DM and IBD.

The innate immune system significantly upregulates TNF-α, a crucial pro-inflammatory cytokine, in individuals with DM and IBD. Infliximab, adalimumab, and certolizumab are three anti-TNF-α monoclonal antibodies often used to treat IBD patients. These antibodies bind to both soluble and membrane-bound TNF-α, effectively blocking its interaction with receptors and inhibiting its activation. A short retrospective study examined the levels of HbA1c, fasting blood plasma glucose, and fasting triglycerides in individuals with rheumatoid arthritis or Crohn’s disease who were using etanercept or infliximab. The study showed that TNF-α inhibitors can enhance glucose control in diabetic patients [138]. Another study discovered that certolizumab treatment enhances insulin sensitivity and reduces IR by inhibiting TNF-α, which promotes a decrease in plasma glucose levels and increases insulin sensitivity [139]. The IL-1β antagonist anakinra has demonstrated the ability to increase blood sugar levels and beta cell production in individuals with diabetes and prediabetes [14]. In an animal rat model, Ozdemir et al.’s findings indicate an improvement in IR, both systemic and local inflammation, and oxidative damage, resulting in a positive change in the pancreas’ histological structure and a decrease in beta cell malfunction [140].

Another study by Liso et al. suggests that anakinra may be a more beneficial therapeutic option for initial nonresponders to anti-TNF-α therapy, therefore offering fresh knowledge and alternative treatment strategies for UC patients [141]. There is a limited amount of research on the role of anakinra in IBD, however, one study provides fresh perspectives and different approaches for treating individuals with UC. Considering that the trial is now in the second testing phase, it is too early to discuss any outcomes at this point [142]. In addition to controlling blood sugar levels, the intestinal hormone GLP-1 also exhibits anti-inflammatory, anti-apoptotic, and antioxidant properties [143]. Potential mechanisms of GLP activity in IBD include promoting the damaged epithelium’s tissue repair, controlling T cell development and functions, controlling innate immune cells such as dendritic and macrophage cells, and lowering pro-inflammatory cytokines. GLPs, unlike corticosteroids and biologics, do not directly suppress the immune system and could be the first-line treatment for IBD [144].

Metformin has both anti-inflammatory and antioxidant properties, and it improves intestinal barrier integrity in both cellular (in vitro) and animal (in vivo) models of IBD. In addition, metformin can restore the gut microbiota in animals with colitis, decreasing inflammation in the intestines [145]. Ke et al.’s study demonstrated new insights into the role of metformin’s mucus-barrier protection and anti-inflammatory effects. Metformin alters the gut microbiota in mice with UC by increasing the number of possible probiotics, such as Akkermansia muciniphila, and balancing out the bacterial population. This implies that this drug could be very potent for UC treatments [146].

Since diet plays a significant role, DM and IBD may result from or be caused by a modified diet linked to dysbiosis. Therefore, maintaining a healthy diet is necessary for controlling both DM and IBD. Diet can modify the microbiome, subsequently impacting various aspects such as lifestyle, quality of life, and clinical symptoms [147]. In individuals with DM, a well-balanced diet that limits carbohydrate intake is beneficial in regulating weight, controlling blood sugar levels, and lowering the risk of complications, including cardiovascular diseases [148]. It is also essential to emphasize the role of vitamin supplementation in the management of these diseases, with particular attention to vitamin D. Vitamin D plays a crucial role in several physiological processes, including immune modulation and inflammation regulation [149,150]. In diabetes, maintaining adequate vitamin D levels could improve insulin sensitivity and glycemic control, indicating a potential role in optimizing metabolic outcomes [151]. In the context of IBD, the ability of vitamin D to modulate the immune system can help reduce chronic inflammation and support mucosal integrity [152]. Therefore, with adequate vitamin D supplementation, it is possible to enhance therapeutic strategies and improve clinical results in people with both DM and IBD. For IBD patients, identifying and avoiding trigger foods, ensuring adequate nutrient intake, and incorporating anti-inflammatory foods can manage symptoms and reduce inflammation. Both conditions benefit from personalized dietary plans and professional guidance to monitor and adapt to individual needs, ultimately improving quality of life and disease management.

## Conclusion

Chronic inflammation and an abnormal immune response involving toll-like receptors (TLRs) and the NLRP3 inflammasome causing tissue damage are underlying factors contributing to the pathogenesis of DM and IBD. Increased intestinal permeability, immunological modulation, metabolic dysfunction, and inflammation are all influenced by gut dysbiosis in both disorders. Although there are evident similarities, further comprehensive investigations, research, and clinical trials are necessary to improve our understanding of the interconnected pathways underlying both DM and IBD for the development of precise and personalized medicines that can successfully target chronic inflammation, improve clinical outcomes, and reduce the incidence of one in the presence of other.

## Key points, Outstanding Questions and Challenges

- **Chronic inflammation as a common driver:** Both DM and IBD share chronic inflammation as a central pathophysiological feature, with significant overlap in their inflammatory pathways.
- **Genetic and environmental influences:** Genetic predispositions and environmental factors such as diet, lifestyle, and infections significantly influence the development of DM and IBD.
- **Disease onset and causality:** Which disease typically develops first, DM or IBD? Is there a causal relationship, or do they arise independently due to shared risk factors?
- **Impact of dysbiosis:** How do specific changes in gut microbiota composition contribute to the progression of DM and IBD?
- **Preventive strategies:** Developing preventive strategies for individuals with a risk of both DM and IBD is challenging due to the multifactorial nature of these diseases.
- **Overlapping therapeutic approaches:** Managing DM and IBD with common therapies is complex because of differing efficacy, side effects, and the need for personalized, coordinated care.
